# Small behavioral adaptations enable more effective prey capture by producing 3D-structured spider threads

**DOI:** 10.1038/s41598-019-53764-4

**Published:** 2019-11-21

**Authors:** Caroline C. F. Grannemann, Marco Meyer, Marian Reinhardt, Martín J. Ramírez, Marie E. Herberstein, Anna-Christin Joel

**Affiliations:** 1 Institute of Biology II, RWTH Aachen University, Aachen, Germany; 2 Museo Argentino de Ciencias Naturales “Bernardino Rivadavia”–CONICET, Buenos Aires, Argentina; 3 Department of Biological Sciences, Macquarie University, Sydney, Australia; 4 Present Address: RWTH Aachen University, Institute of Biology II, Worringerweg 3, 52074 Aachen, Germany

**Keywords:** Animal behaviour, Biomechanics

## Abstract

Spiders are known for producing specialized fibers. The radial orb-web, for example, contains tough silk used for the web frame and the capture spiral consists of elastic silk, able to stretch when prey impacts the web. In concert, silk proteins and web geometry affects the spider’s ability to capture prey. Both factors have received considerable research attention, but next to no attention has been paid to the influence of fiber processing on web performance. Cribellate spiders produce a complex fiber alignment as their capture threads. With a temporally controlled spinneret movement, they connect different fibers at specific points to each other. One of the most complex capture threads is produced by the southern house spider, *Kukulcania hibernalis* (Filistatidae). In contrast to the so far characterized linear threads of other cribellate spiders, *K. hibernalis* spins capture threads in a zigzag pattern due to a slightly altered spinneret movement. The resulting more complex fiber alignment increased the thread’s overall ability to restrain prey, probably by increasing the adhesion area as well as its extensibility. *Kukulcania hibernalis*' cribellate silk perfectly illustrates the impact of small behavioral differences on the thread assembly and, thus, of silk functionality.

## Introduction

Spiders are renowned for the production of specialized fibers. Especially their tough dragline silk has attracted the attention of many researchers to date^1–5^. In addition to dragline silk, spiders produce several different types of silk with properties relative to their application^6^. In an orb web, for example, the tough dragline silk is used for the production of the radial frame, but the capture spiral consists of more elastic silk, which is able to stretch rather than brake when prey is impacting the web^7^. For example in *Araneus diadematus*, the capture thread has a seven times higher extensibility compared to dragline silk^8^.

Several studies have shed light on how different structural features of the spider web influence the mechanical performance of the overall web^4,9,10^. Additionally, it is known that the mechanical properties of silks are the result of folding in spidroins, the silk fiber’s proteins^11–13^. However, little attention has been paid to the mechanical impact of the way spiders process fibers to form more complex structures, with notable exceptions, such as the attachment discs of spiders, the looped ribbon silk of *Loxosceles laeta* and the cribellate frame thread of *Progradungula otwayensis*^14–16^. In general, the most complex processing of fibers is performed by this paraphyletic group of cribellate spiders, which includes aside from *P. otwayensis* also e.g. Uloboridae and Filistatidae. In contrast to the typical orb-web building ecribellate spiders, using viscid silk as glue to capture prey, cribellate spiders have a dry adhesive capture thread consisting of nanofibers. The viscid glue is assumed to be the derived adhesive mechanism.

Depending on the cribellate species, several thousand nanofibers (Ø 10–30 nm) are assembled with a number of larger fibers into a complex capture thread^17–19^. Most studies so far have tried to understand the capture thread production of cribellate uloborid spiders, as they produce very simple structured capture threads^17,20,21^. These threads consist of two parallel axial fibers as core fibers, produced by pseudoflagelliform spigots^17,20^, absorbing most of the energy during prey capturing^22^. These axial fibers are surrounded by a sheath of the nanofibers emerging from the cribellate spigots^17^. To connect all fibers the nanofibrillar mat is probably sewed to the axial fibers with paracribellate fibers from the paracribellate spigots by a timed movement of the spinnerets during capture thread production^17,20^. The connection between axial fibers and the paracribellate fibers is established during the retraction of the combing leg, when the abducted median spinnerets and the adducted posterior spinnerets move their spigots in close proximity to each other^17^. The final puffy structure of the thread is produced by a comb on the metatarsus of the fourth leg, the calamistrum, probably influencing the protein conformation when brushing over the freshly extracted nanofibers^17,23^. As this is a rather linear alignment of fibers in the thread, this thread assembly can abstractly be described as 2D-structure.

The hypothesized function of the different fibers in capturing prey is rather simple: The nanofibers are assumed to be the adhesive of this capture thread and are embedded in the cuticular waxes covering insects, similar to the wax-wick interaction of a burning candle^24^. Additionally, van der Waals, and in some threads hygroscopic forces play a role in the adhesion of cribellate capture threads, at least on artificial surfaces^25^. The axial fibers are on the other hand necessary to provide the strength of the thread, able to withstand the forces of struggling prey^22^. An energy transfer is probably facilitated by the paracribellate fibers. Mechanical analyses showed that cribellate threads are less extensible than the capture threads of ecribellate spiders, which are using viscid glue instead of nanofibers (~350% based on eight cribellate species compared to ~ 560% from 14 ecribellate species)^16,26–28^. However, if one excludes the elasticity data of uloborids’ cribellate capture threads, only two cribellate species (*P. otwayensis* and *Deinopis spinosa*) remain showing a much greater extensibility (~680%).

**Figure 1:**
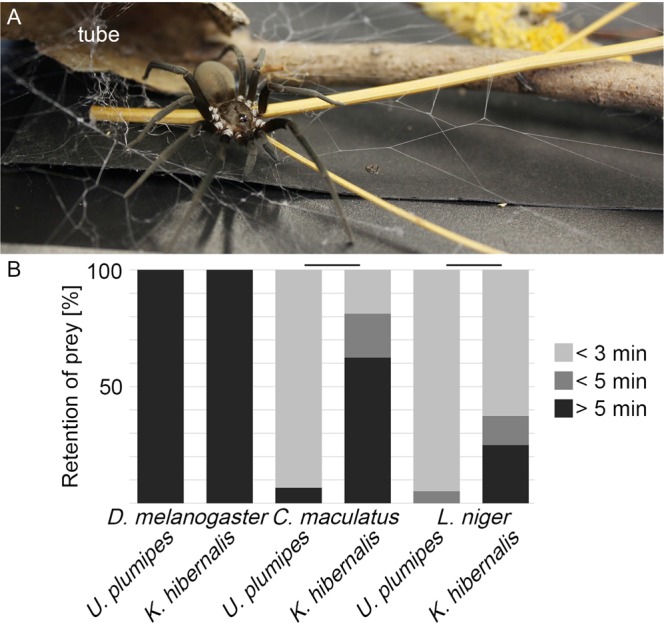
(**A**) *Kukulcania hibernalis*. (**B**) Time a prey item needs to escape a single capture thread of either the smaller uloborid *Uloborus plumipes* or the larger filistatid *K. hibernalis*. The bar above two columns means that these two are significantly different (G-test, p < 0.005).

In contrast to uloborids, almost all other cribellate spiders have much more complex cribellate threads, including more fiber types, and many aspects of their functionality as well as their production process are not yet fully understood^18,19,29^. A recent study on *P. otwayensis* recorded massive differences in elasticity as well as adhesion as soon as the structure of the thread changes, even within one species^16^. One example of building a very complex cribellate thread is the southern house spider, *Kukulcania hibernalis* (Filistatidae; Fig. 1A). Though this is a spider with many basal characters, published pictures of their capture threads show a very sophisticated looped 3D structure, which may be linked to its functionality by increasing adhesion^23,30,31^. The ultrastructure of cribellate threads suggests that filistatid threads consist of one, or maybe more, undulating fibers (sometimes also called reserve wrap), possibly one or more axial fibers and flattened, non-nodular cribellate nanofibers^18,19,32,33^. This cribellate thread is attached periodically to a thick foundation line^18^. It is assumed that the puffy structure is missing from the threads of these spiders^18^. All-in-all, there are many question regarding the thread (ultra)structure and thread production that need to be answered (besides the leg’s combing position^34^). It is even unknown where each fiber originates from.

Because structure can massively influence functionality, the existing vague descriptions are insufficient to understand how the thread assembly functions. For example, the adhesive properties of non-nodular nanofibers do not increase with increased humidity, whereas they do in nodular cribellate fibers that are produced by the majority of cribellate spiders^25,35^. Additionally, the adhesive properties of *K. hibernalis*’ capture threads do not match the excessive size of the spider (~19 mm body length), requiring more or larger prey^36^. The adhesive strength of cribellate threads is positively correlated with the number of nanofibers^36^ but *K. hibernalis* has only about 4300 cribellate spigots, and so produces fewer nanofibers compared to the relatively smaller sized uloborid *Uloborus plumipes* (~5 mm body length) with 5100 spigots (pers. obs., Opell^31^).

Web-building uloborids indeed have a cribellum with many more spigots compared to body size than other cribellate spider^36^. This has been linked to their aerial orb web and the prey capture. *Kukulcania hibernalis*, on the other hand, builds radiating substrate webs^30^. Though *Uloborus* and *Kukulcania* are very different in body size, web-building behavior and from a phylogenetic point of view, the quality of their adhesive capture threads is believed to be mainly linked to the number of cribellate fibers. Since we know that the mechanical performance is influenced by the threads’ structure, we asked: How can the much larger *K. hibernalis* capture enough prey, if its adhesive strength is predicted to be inferior to that of *U. plumipes*? Is a looped 3D structure enough? Or are there some hidden structural features that enable the capture of larger prey? If so, what are the inherited behavioral differences associated with the production? To answer these questions, we evaluated the ability of *K. hibernalis*’ threads to restrain prey and combined these data with the analysis of their capture thread assembly. As a well-studied reference species, we used the 2D thread producing *U. plumipes*.

## Results

### Retention of prey

To understand how the much larger *Kukulcania hibernalis* can capture enough prey, despite the predicted lower adhesive strength compared to *Uloborus plumipes*, we compared the prey retention capacity of *K. hibernalis*’ and *U. plumipes*’ capture threads. The capture threads of *K. hibernalis* were significantly better at retaining beetles and ants with fewer prey escaping compared to *U. plumipes* (Fig. 1B; beetles, *K. hibernalis*: n = 16 and *U. plumipes*: n = 15, ant, *K. hibernalis*: n = 16 and *U. plumipes*: n = 19; flies, *K. hibernalis*: n = 17 and *U. plumipes*: n = 12; G-test, p < 0.005). Vinegar flies could not struggle free of any of the threads from either species. We next investigated how the thread of *K. hibernalis* can be stronger, despite being theoretically inferior.

### Analysis of the 3D thread structure

**Figure 2:**
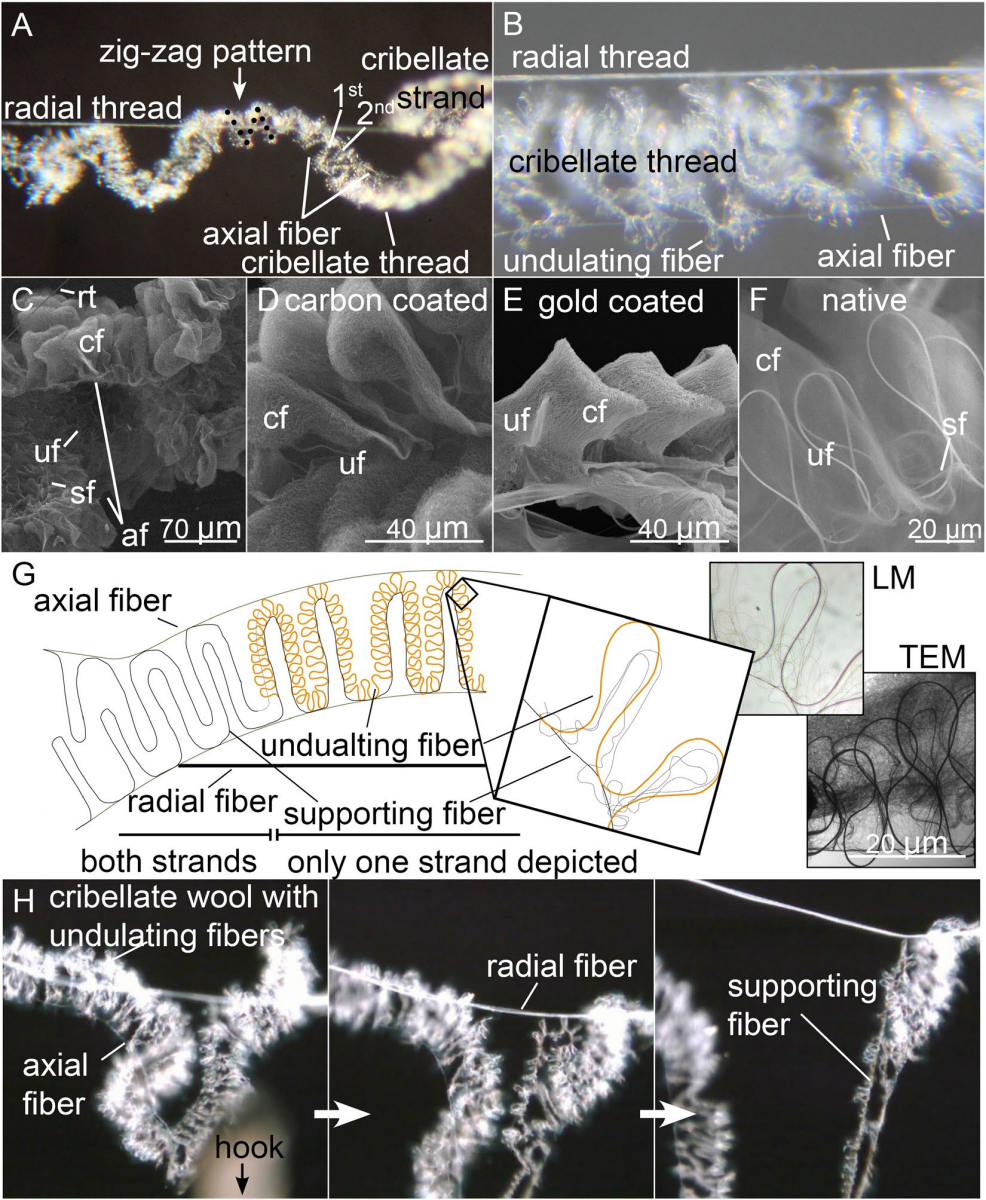
(**A,B**) Light microscopic images of capture threads of juvenile spiders, showing the general structure of the cribellate thread. (**C**) Insight view of the cribellate thread, showing the axial fibers (af) at the rim of the zigzag aligned cribellate fibers (cf). rt: radial thread, sf: supporting fibers, uf: undulating fibers. SEM, carbon coated sample. (**D–F**) Close up of the puffs, showing the different types of fibers visible with different coating techniques in the SEM. (**G**) Simplified model of the internal structure of the capture thread of *K. hibernalis*. On the left side is depicted how both strands are interlocking to form one thread, and on the right side only one of two parallel strands were drawn for simplification. The cribellate fibers, accompanying the undulating fibers, are not depicted here. Original pictures are added to highlight the assembly of the different fibers in the puffs (upper right: light microscopy; lower right: TEM). (**H**) Splitting capture thread when pulling at the thread (here: down). Please note on the left side the different states of disruption: first the axial fibers break and then the supporting, before finally the complete thread ruptures (Movie 1).

*Kukulcania hibernalis* builds tubes with capture threads radiating from their hiding place (Fig. 1A). Therefore, as the spider left the retreat, it trailed behind a radial thread consisting of six fibers. When the spider turned to walk back to its retreat, it extracted cribellate silk, which it laid down onto the radial thread, attaching the cribellate thread at irregular intervals (see also Eberhard^34^ or Lopardo and Ramirez^37^). When we pulled at the cribellate thread to separate it from the radial thread, it became obvious that it was fixed to the radial thread only at two attachment points. The intervening structure was looping randomly around the radial thread. Sometimes, the cribellate thread was not fixed along the radial thread, but between two of these. This was especially true for silk near the retreat of the spider. There, the looped structure was absent, which facilitated the observation of the hierarchical structure of the thread more easily (Fig. 2).

A closer examination of the cribellate thread revealed, *K. hibernalis* was not producing one linear cribellate thread like uloborids. With its divided cribellum it produced two parallel bunches of cribellate fibers, arranged in puffy structures. Despite having a divided cribellum, these spiders did not split both bunches in two separated capture threads, but both were arranged side by side in one thread (Fig. 2A,B,G,H). Each of the two cribellate bunches was accompanied by a thick, undulating fiber with oval profile and three thinner undulating fibers woven together with the larger one (Fig. 2D–G). Additionally, smaller fibers were randomly imbedded in the bunch. With a similar thickness to the smaller undulating fibers, a supporting fiber built a straight base line of each bunch (Fig. 2F–H). These cribellate strands, composed of cribellate, undulating and supporting fibers, are comparable to the 2D capture threads of uloborids, though more complex. However, they were again organized in a zigzag pattern, with eight to 15 of the puffy structures spun between two axial fibers (Fig. 2). When we pulled vertically at the looped capture thread, this pattern split step by step, breaking the axial fibers first and then the supporting ones (Fig. 2H, Movie 1). This enabled a capture thread extensibility more than 26x its own length before breaking the entire thread.

### Origin of silk fibers

**Figure 3:**
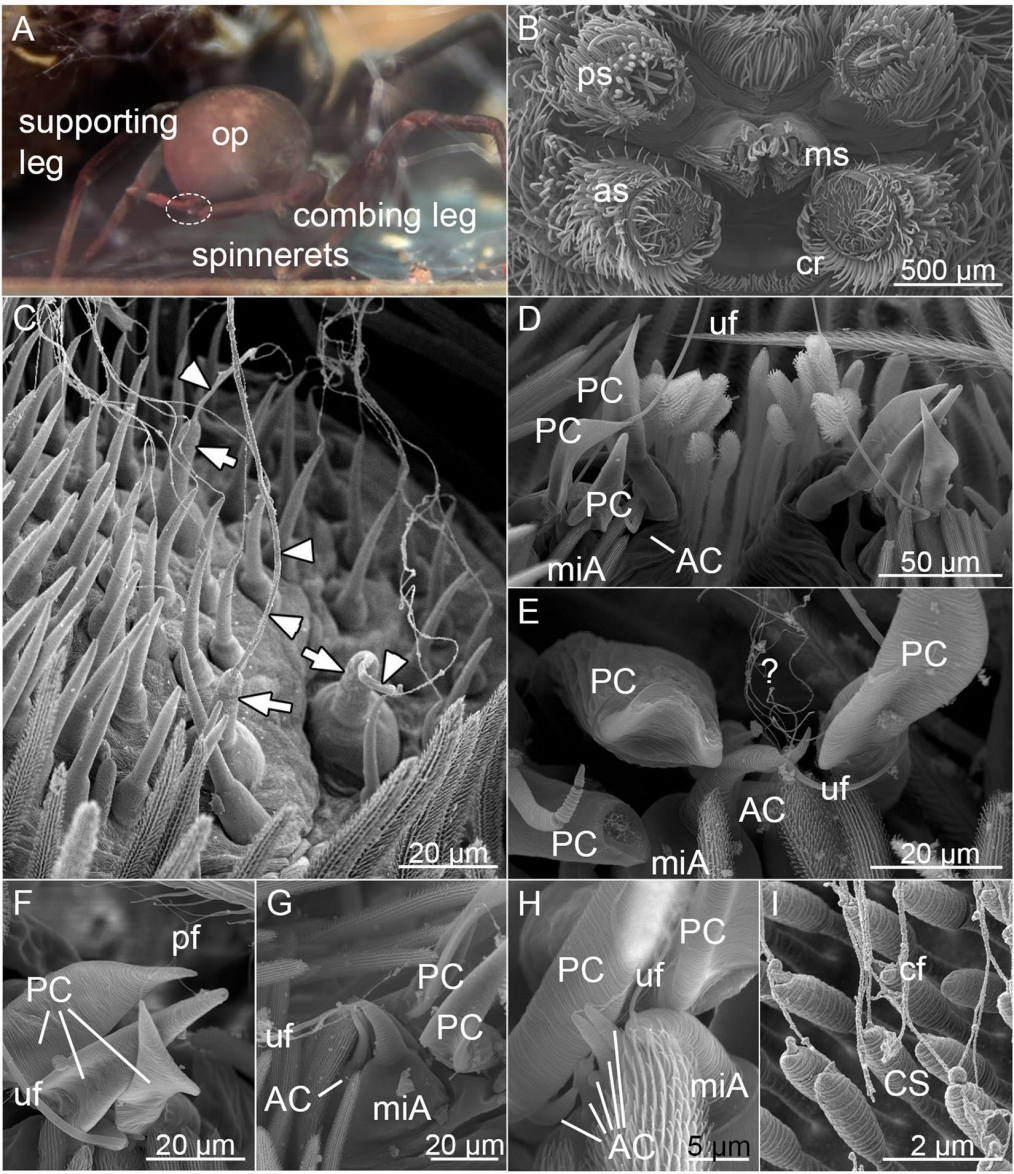
(**A**) *K. hibernalis* during capture thread production. (**B**) Overview of spinnerets, anterior to bottom. as: anterior spinneret, cr: cribellum, ms: median spinneret, ps: posterior spinneret. (**C**) Silk of radial thread emerging from anterior spinnerets. Arrows: major ampullate spigots, triangles: major ampullate fibers. (**D**) Both median spinnerets, showing the positions of the paracribellate spigots (PC), the minor ampullate gland spigot (miA) with emerging undulating fiber (uf) and the aciniform gland spigots (AC). (**E**) Close-up of the median spinnerets showing again the paracribellate spigots (PC; tip is broken off in two cases), a minor ampullate gland spigot (miA) with emerging undulating fiber (uf) and several smaller fibers of unknown origin, maybe from the aciniform gland spigots (AC). (**F**) Median spinneret, with focus on the three paracribellate spigots (PC) and their emerging paracribellate fibers (pf). Please note, that the fibers from the aciniform gland spigots and the paracribellate spigots have similar diameter and thus cannot be distinguished in the final thread. uf: undulating fiber. (**G**) Close-up of the median spinnerets showing the undulating fiber (uf) emerging from the minor ampullate gland spigot (miA). Please note the smaller fibers, emerging from the aciniform gland spigots (AC), accompanying the uf. PC: paracribellate spigot. (**H**) Fibers emerging from the aciniform gland spigots (AC). miA: minor ampullate gland spigot, PC: paracribellate spigot, uf: undulating fiber. (**I**) Detail of the cribellum, showing the cribellate spigots (CS) and emerging cribellate fibers (cf). B to I: SEM images.

The six thicker radial fibers were traced to three major ampullate gland spigots on each anterior spinneret (Ø 1.8 ± 0.3 μm, n_1_ = 6; Fig. 3C). Otherwise, the anterior spinnerets were not involved in the capture thread production. The oval undulating fiber ( = reserve warp) emerged from the minor ampullate gland spigot, one on each median spinneret (larger diameter 2.0 ± 0.2 µm, n = 2; Fig. 3D to H). Three smaller undulating fibers, woven together with the oval undulating fiber, had an unremarkable morphology with similar diameter to the supporting fiber (0.4 ± 0.1 µm, n = 3). They were tentatively matched to the three paracribellate spigots on each median spinneret (Fig. 3F,G), as their number and diameter matched the number of spigots as well as the diameter of fibers emerging from them (0.2 ± 0.0 µm, n = 2; see Supplementary Table S2). Occasionally, the aciniform spigots of the median spinnerets were emitting silk of a similar diameter (0.3 ± 0.1 µm, n = 2; Fig. 3F,H). They might be producing the randomly aligned smaller fibers. We were not able to determine a suitable spigot on either spinneret producing the supporting fiber, nor could we trace the origin of the axial fiber with our fixation. Cribellate nanofibers were the thinnest (Fig. 3I), with their origin being the cribellate spigots on the cribellum.

### Sophisticated thread structure through complex production

To understand how the spider produces such a sophisticated structure, we analyzed the capture thread production of *K. hibernalis* (Fig. 3A). With a frequency of 12.2 ± 0.4 Hz (n = 4), the spider moved the combing leg in an ellipsoid manner over the spinnerets (Fig. 3B), lowering the leg during its posterior movement. After reaching the most posterior position, the leg reversed its movement and enhanced its velocity 1.9 ± 0.6 times (n = 3). Thread production always started with the combing leg anterior to the spinnerets and stopped in the most posterior position of the leg during one combing cycle (i.e. ellipsoid movement of combing leg). This observed behavior resembled the one of other cribellate species described so far, albeit faster (12 Hz vs 8 to 10 Hz) (Table 1)^17,21^.

**Table 1:** Comparison of behavioral data.

Family	Uloboridae		Filistatidae
Species	U. plumipes^*a^	Z. geniculata^*b^	*K. hibernalis*
Combing frequency [Hz]	10.4 ± 0.1 n = 2	8.7 ± 0.4 n = 3	12.2 ± 0.4 n = 4
Relation of velocity of posterior leg movement to anterior leg movement [times slower]	3.7 ± 1.2 n = 3	3.1 ± 0.9 n = 3	1.9 ± 0.6 n = 3
Combing position^*c^	Type 2		Type 1
Start capture thread production	Combing leg anterior to spinnerets		
Stop capture thread production	Combing leg posterior to spinnerets		
Lowering of metatarsus	Posterior leg movement		
Abduction posterior spinnerets	Posterior leg movement		1.1 ± 0.0 Hzn = 3
Abduction median spinnerets	Anterior leg movement		24 Hzn_1_ = 2

^*a^Data from Joel, *et al*.^17^, ^*b^data from Joel, *et al*.^21^, ^*c^definition after Eberhard^34^.

However, when characterizing the movement of the spinnerets, major differences to the so far described cribellate thread production process could be determined: the median spinnerets were moving twice during one combing cycle, with a frequency of 24 Hz (n_1_ = 2) (see Supplementary Table S1, Fig. S1 and Movie 2–4). Likewise, two loops of undulating fibers corresponded to one combing cycle, with one loop building one puff. So, we assume that two puffs constitute one structural unit in *K. hibernalis*. The posterior spinnerets showed an asynchronous movement relative to each other and moved much slower than the combing leg with a frequency of 1.1 ± 0.0 Hz (n = 3). In addition, they did not perform a steady movement, but were abducted most of the time during one cycle, and were only very shortly adducted.

## Discussion

### Capture thread production in *Kukulcania hibernalis*

The filistatid *K. hibernalis* may have many primitive characters and represents an early branch in the phylogeny of true spiders^38^. Nevertheless, this spider produces highly sophisticated capture threads, structurally more complex than in the modern uloborids, as well as other cribellate spiders^30,36^. These complex capture threads are key to explaining why and how such a large spider can capture equally large prey, though theoretically with capture threads of inferior adhesive strength compared to other cribellate spiders.

**Figure 4:**
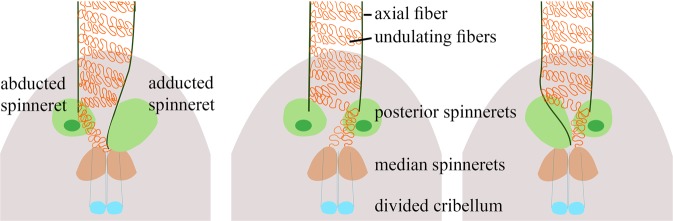
Model of the capture thread production of *K. hibernalis*, showing the influence of the slower and asynchronous movement of the posterior spinnerets on the thread assembly. Please see Movie 2–4 for original data of the spinneret movement.

Several features of the capture thread production seem to be conserved between filistatids and uloborids. For example, the calamistrum processes the fibers during the posterior movement of the combing leg (Table 1). However, in contrast to uloborids^17,21^, two undulating substructures (resembling the puffy structures in capture threads of other spiders) are produced by *K. hibernalis* during one stroke of the combing leg. This matches the frequency of the median spinneret movement, twice as fast as the combing leg, suggesting they are responsible for these structures. Tracing the undulating fibers to the minor ampullate gland spigots of the median spinnerets confirms this hypothesis. Additionally, *K. hibernalis* does not produce a linear 2D capture thread but its cribellate strands are spun between two axial fibers in a zigzag pattern. In general, it is assumed that a close proximity of spigots leads to contact and thus a connection between the emerging fibers^17,39^. In a linear thread alignment like in uloborids, the cribellate fibers are regularly sewn onto the axial fibers by the paracribellate fibers^17,21^. This sewing is facilitated by inducing contact between the paracribellate spigots (median spinnerets) and the pseudoflagelliform spigots (posterior spinnerets), that typically produce the axial fibers in other cribellate spiders^17,20,40^. In uloborids, the spinneret movement is synchronized to the combing leg movement, producing one of these connections during one stroke of the leg. To produce a zigzag pattern of the cribellate strands between two axial fibers, there has to be reduced contact between the paracribellate spigots and the pseudoflagelliform spigots. Therefore, either the median spinnerets have to stay adducted or the posterior spinnerets are abducted for a longer period. In fact, the posterior spinnerets were abducted for most of the time and each moved about 12 times slower than the combing leg and 24 times slower than the median spinnerets. Combing their speed with their asynchronous movement, it matches the 12 substructures of the cribellate wool produced between the two axial fibers perfectly. Thus, we argue that the zigzag pattern is due to the slower movement of the posterior spinnerets resulting in fewer connections between axial fibers and cribellate mat (Fig. 4).

We were not able to determine how the cribellate strand, together with the undulating fibers, is incorporated into the supporting fiber, mainly because we were not able to determine the spigot from which the supporting fiber emerges. A closer examination, evaluating the influence of position and size of spigots on the spinnerets, could help to resolve this issue, especially as there is no obvious spinneret movement matching the position of the supporting fiber in the final thread.

### Impact of sophisticated structure on capture thread properties

The complex fiber arrangement and the associated more complex production of the thread pays off for *K. hibernalis*. This capture thread structure compensates for its theoretically inferior adhesive properties, due to having non-nodular and fewer cribellate fibers. Compared to uloborids, *K. hibernalis* threads can retrain prey for longer. We assume there to be two reasons: 1. Typically the adhesion forces are matched with the number of available nanofibers extracted from the cribellum^36,41,42^. Due to the zigzag pattern, the “number”, i.e the length, of the nanofibers in contact with the prey increases. This would follow the reasoning of Michalik, *et al*.^16^, arguing a greater adhesion force in processed cribellate capture threads, compared to the same species cribellate frame threads. 2. The fibers in the capture thread have different breaking strengths, which lead to tearing off single fibers without disrupting the complete thread. This results in a greater extensibility of the thread, much higher than observed in any of the silks of e.g. the ecribellate spiders *Araneus diadematus* or *Argiope trifasciata*^12,43^ as well as the so far characterized cribellate capture threads of Deinopidae, Uloboridae and Gradungulidae^16,22^. In *Progradungula otwayensis* a similar argument has been made, suggesting a more complex structure consisting of undulating and axial fibers leading to a greater elasticity^16^. In case of *K. hibernalis*, though, the extensibility is destructive and thus of course not reversible. However, *K. hibernalis* is not an orb-web spider and captures walking rather than flying prey. Thus, as long as the prey stays in contact with the web, even via to a very stretchy and deformed thread, the spider can locate and subdue it. High extensibility of threads can hence not only be achieved by their stretching properties and by elastic deformation of the involved fibers, but can be behaviorally achieved by a sophisticated 3D arrangement. The difference in breaking strength of the involved fibers most likely mirrors their amino acid composition, which is undescribed for all silks involved in *K. hibernalis* capture thread.

## Conclusion

Providing an insight into the capture thread structure as well as the spider’s behavior, our results confirmed several features of the cribellate capture thread production conserved even in distantly related species. Instead of having to create a new model for cribellate capture thread production, the southern house spider, *Kukulcania hibernalis*, perfectly illustrates the impact of small behavioral differences on the thread assembly and thread functionality. The final more complex capture thread structure is advantageous for prey retention: while capture thread production takes longer per mm due to a zigzag pattern instead of a linear alignment, this structure helps the spider to restrain prey for longer. This is probably caused by a mixture of increased adhesion forces as well as its enormous extensibility due to breaking and disentangling of single fibers without breaking the complete thread.

## Methods

### Study animals

Southern house spiders, *Kukulcania hibernalis* (Hentz, 1842), were raised separately under elevated room temperature (~26 °C), normal room humidity (~30%) and northern European diurnal rhythm. Once a week, spiders were fed with juvenile *Acheta domestica* or *Callosobruchus maculatus*. The spider often ignores smaller prey items, like *Drosophila melanogaster*, suitable to feed smaller spiders. Water was provided once to twice per month by applying droplets near the burrow. Wetted threads in proximity to the burrow were not used for the analysis.

*Uloborus plumipes* (Lucas, 1846) were raised under room temperature (~21 °C), room humidity and northern European diurnal rhythm. Once a week, spiders were fed with *D. melanogaster*. Water was provided once to twice per month by sprinkling the web. Threads of the sprinkled webs were not used for analysis.

### Prey retention

Black garden ants (*Lasius niger*), cowpea weevils (*C. maculatus*) and vinegar flies (*D. melanogaster*) were used to determine the retention time of prey. The insects were gently picked up with featherweight forceps and placed on a single capture thread spun between two arms of a metal wire (distance ~7 mm), so the insect was not able to touch neither ground nor wire (Movie 5). If the insects’ movement were curtailed by the procedure, the trial was discarded to eliminate bias due to distorted behavior. In successful trials, the time until escape was measured. Spiders typically react directly to living prey within the web. Therefore, the relevant time is ‘<3 min’ (prey might be able to escape) and ‘<5 min’ (prey likely to be captured). All trials above ‘>5 min’ would result in prey definitely been captured by the spider.

### Analysis of thread structure

Threads were taken from the web with two stripes of conductive foil (distance <5 mm) on a sample holder. The samples were observed after coating with carbon or gold or without any further preparation (native) in a scanning electron microscope (SEM 525 M, Philips AG). Please note that gold coating leads to artefacts in the thread’s structure, but some structural components are more easily discriminable using this method^23^.

Thread structure was also observed using transmission electron microscope (EM 10, Carl Zeiss AG) or with a light microscope.

### Origin and measurement of silk fibers

To trace the origin of individual fibers we fixed the silk to the spigots by dripping them with hot molten paraffin during the spinning process, as in Peters^44^, and later dissolved in xylol and then to EtOH in a graded series, critical point dried, coated with gold-palladium and observed in a standard SEM (FEI-XL30TMP) or a field emission SEM (Zeiss Supra). Measurements of silk fibers were done in Adobe Photoshop using the scale provided by the electron scanning microscope, averaging five measurements per fiber. Raw data are presented in the supplement (Table S2). Cribellate nanofibers were not measured with this technique, since the coating is close to the scale of measurement and their origin is without doubt.

### Thread extensibility

To test the thread’s behavior during stretching, threads were taken from the web with two parallel metal wires (twisted zinced paper clips) with a distance of approximately 0.7 cm. A metal hook, controlled with a micromanipulator (M331R, Märzhauser Wetzlar GmbH & Co. KG), clasped a loop of the thread and the loop was drawn away from the radial thread. The behavior of the thread was documented with a digital microscope (VW-9000C; Keyence Cooperation).

### Analysis of spinneret and leg movement

*Kukulcania hibernalis* nocturnal web building behavior was observed under red light using a webcam (Logitech HD Webcam C270, Apples; Software: Logitech Webcam Software Version 2.5.1). To analyze leg and spinneret movement, the spider was placed into a smaller box (~0.2 l) with roughened substrate for walking, wooden sticks for web support and a piece of dried leaf for hiding. The spider was either recorded from a side view, using a binocular microscope with 10 to 40× magnification and an attached digital single-lens reflex camera (50 fps; EOS 550D, Canon). Otherwise, the boxes were brought into focus of a high-speed video recording microscope (VW-9000C) and recordings were performed with up to 1000 fps and 100× magnification. Online available recordings were used to assess the general validity of our results, like frequency or the movement of the posterior spinnerets (see Supplementary Table S1). Joel, *et al*.^21^ provides additional details about using online available recordings for these kinds of measurements.

All videos were analyzed using Keyence VW-9000 Motion Analyser (Version 1.4.0.0) or a custom-written script (Matlab R2014b Version 8.0.0.783, The MathWorks Inc.) for manual tracking the spinneret and leg movement (lateral view). This manual tracking facilitates a more precise temporal and spatial resolution. In recordings where the thread was visible, the spinneret movement was matched to the produced thread structure. Additionally, the number of undulating structures within one zigzag-pattern were counted in SEM pictures and matched to the different spinneret movement frequencies.

### Statistical analysis

Data are presented as mean ± standard deviation (SD), with ‘n’ representing the number of different individuals. As several recordings/data points were collected per spider, we calculated the mean for each spider individually before calculating the overall mean. If data of only one spider were available (noted as ‘n_1_’), the SD refers to the intra-individual variance and ‘n_1_’ indicates the number of data points. To evaluate differences in the retention time, a G-test with two degrees of freedom was performed. Significance was assumed, if p < 0.05. Calculations were performed with Microsoft Office Excel 2013.

## Supplementary information

Supplement

Movie 1 Capture thread disruption

Movie 2 Kukulcania_lateral_4.17x

Movie 3 Kukulcania_ventral_4.17x

Movie 4 Kukulcania_ventral_33.3x

Movie 5 Retention experiment

## Data Availability

Data supporting this article have been uploaded as electronic supplementary material.
