# Supplementary material for: Small behavioral adaptations enable more effective prey capture by producing 3D-structured spider threads
Source: Sci Rep. 2019 Nov 21;9:17273. doi: 10.1038/s41598-019-53764-4 (PMC6872738; doi:10.1038/s41598-019-53764-4)
Supplement: Supplementary file 1 — Supplement [file 41598_2019_53764_MOESM1_ESM.pdf]

Supplemental data

Small behavioral adaptations enable more effective prey capture by producing 3D-structured spider threads

Caroline C. F. Grannemann[1], Marco Meyer[1], Marian Reinhardt[2], Martín J. Ramírez[2], Marie E. Herberstein[3] and Anna-Christin Joel[1, 3*]

1: Institute of Biology II, RWTH Aachen University, Aachen, Germany

2: Museo Argentino de Ciencias Naturales “Bernardino Rivadavia” -- CONICET, Buenos Aires, Argentina

3: Department of Biological Sciences, Macquarie University, Sydney, Australia

Corresponding author (*): joel@bio2.rwth-aachen.de

Tab. S1: Online available clips of the cribellate silk production in *K. hibernalis*. If the same spider (i.e. same uploader and same background) is in several available recordings, we clustered the data accordingly. Please note, that we are not the uploader of these movies. However, they nicely proof our results are reproducible. *: juvenile spider.

**Link**	**Spider**	**Date accessed**
https://www.youtube.com/watch?v=HP8KkSKBYYc	1	16/01/2016
https://www.youtube.com/watch?v=HvcNdInCCr0	2	16/01/2016
https://www.youtube.com/watch?v=vzBjUd5QJR0	2	05/01/2017
https://www.youtube.com/watch?v=TbHc_4HpKcE	2	31/01/2017
https://www.youtube.com/watch?v=1X6120WlV9Q	3*	31/01/2017
https://www.youtube.com/watch?v=hh8uUBiF6J8	3*	31/01/2017

Tab. S2: Raw data of fiber diameter measurements (average of five measurements per fiber).

**Spigot/Fiber**	**Spider**	**Ø [µm]**
Big, oval undulating fiber emerging from the minor ampullate spigots	1	1.74
	2	2.07
	2	2.19
Smaller undulating fibers	1	0.17
	2	0.18
	2	0.16
	3	0.19
	3	0.20
Fibers emerging the paracribellate spigots	1	0.23
	1	0.20
	2	0.16
Fibers emerging the aciniform spigots	1	0.22
	1	0.35
Supporting fiber	1	0.25
	2	0.26
	2	0.30
	3	0.46
	3	0.49
Fibres emerging from the major ampullate gland spigots	1	2.08
	1	1.44
	1	1.89
	1	1.37
	1	1.81
	1	2.12

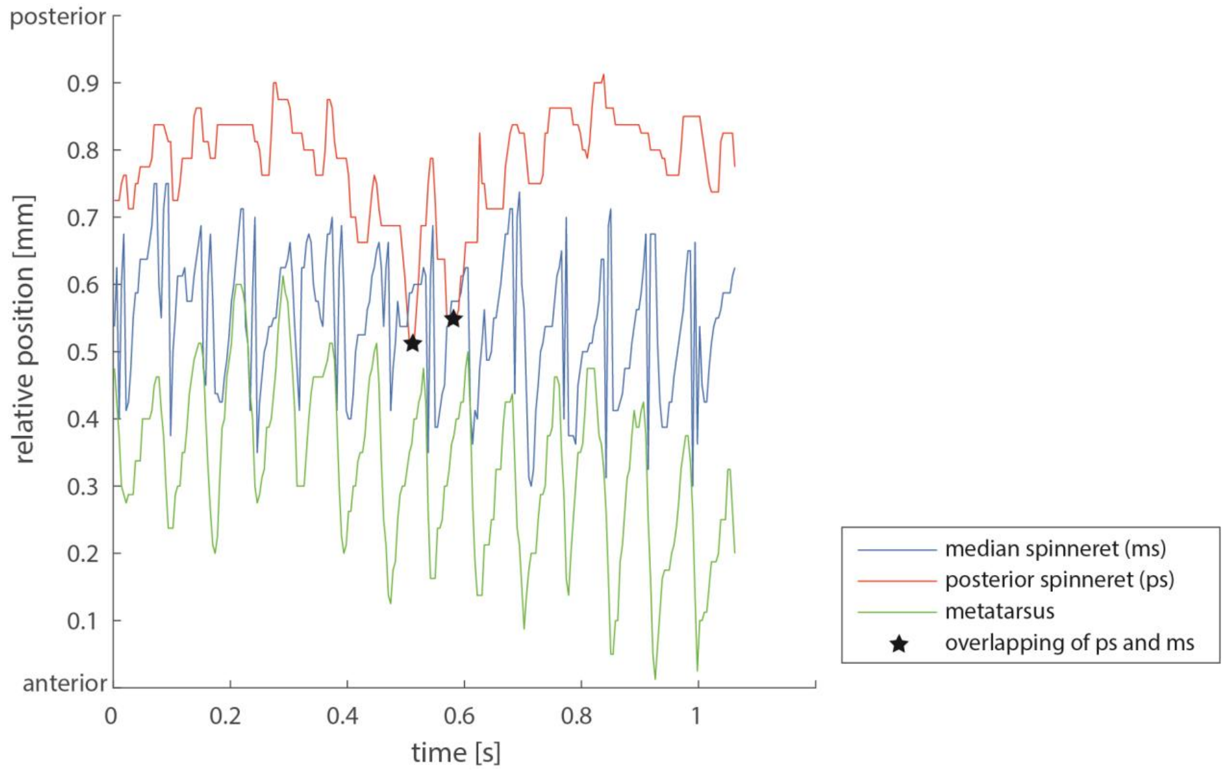

Fig. S1: Data of lateral tracking of posterior (red) and median (blue) spinneret movement, as well as the combing movement of the calamistrum bearing metatarsus (green). Please note that the data for movement in the sagittal plane over time are depicted here.

Description of supplemental movies:

Capture thread disruption

Pulling with a hook at a cribellate thread of *K. hibernalis*. This enables to see the general 3D structure of the thread and how the different fibers influence thread stability. Video corresponding to Fig. 2H. Speed up 16 times.

Kukulcania_lateral_4,17x

Capture thread production in *K. hibernalis*, lateral view, slowed down 4.17 times.

Kukulcania_ventral_4,17x

Capture thread production in *K. hibernalis*, ventral view, slowed down 4.17 times.

Kukulcania_ventral_33,3x

Capture thread production in *K. hibernalis*, ventral view, slowed down 33.3 times.

Retention experiment

Video showing the procedure of placing an insect (here: *D. melanogaster*) into a capture thread (here: *U. plumipes*).
